# Safe surgical training: evaluation of a national functional endoscopic sinus surgery model simulation course using the Kirkpatrick evaluation model

**DOI:** 10.1007/s11845-023-03309-6

**Published:** 2023-02-17

**Authors:** Sarah Louise Gillanders, Alison McHugh, Peter D. Lacy, Mona Thornton

**Affiliations:** 1 Royal College of Surgeons, Dublin, Ireland; 2 Beaumont Hospital Dublin, Dublin, Ireland; 3 Royal Victoria Eye and Ear Hospital, Dublin, Ireland

**Keywords:** Endoscopic, FESS, Simulation, Sinus, Surgery, Training

## Abstract

**Background:**

Simulation is a rapidly developing field in modern undergraduate skills education and postgraduate surgical training.

**Aim:**

We aim to evaluate simulation training as a tool for higher surgical training in functional endoscopic sinus surgery (FESS) using the Kirkpatrick evaluation model.

**Methods:**

This was a prospective cohort study in which a qualitative survey and multiple-choice questionnaire were distributed to otolaryngology trainees pre- and post-FESS training course using simulation models. Participants’ reactions and interpretations of the models were assessed. Pre- and post-simulation knowledge and subjective skills were assessed.

**Results:**

A total of 21 trainees completed the course. Trainees reported simulation models to be accurate representations of human anatomy 95% and easy to use 90%. There was an improvement in anatomical 54 to 62% (*Z* = 76, p0.03) and procedural 65 to 72% (*Z* = 87, p0.03) knowledge overall.

**Conclusion:**

Simulation training is an effective method of postgraduate education. This has been particularly useful following reduced operative exposure in the COVID-19 era.

## Introduction

Simulation is a rapidly developing field in modern undergraduate and postgraduate education. An increased focus on patient safety has left a deficit in training when compared to the old adage “see one, do one, teach one.” European working time directives, limited working hours, and reduced theatre access has necessitated additional training methods from the traditional apprentice model [1]. The use of cadaveric courses and dissection labs was promoted as a safe way to practice new skills without any negative implications for patient safety. Specifically, fresh frozen cadaveric training has been the foundation of FESS training in the past 2 decades, allowing surgical trainees and consultants alike to practice new skills without any negative implication for patient safety. However, these methods are not without limitations. Access to specimens, logistics of running such courses, and cost are limiting factors to cadaver-based training [2], worsened by the recent COVID-19 pandemic. Specifically for FESS, the use of fresh-frozen tissue is preferable to formaldehyde preserved or isolated bony specimens. However, standardisation of fresh specimens is impossible due to variable anatomy and presence/absence of pathology. Recent advances in many surgical specialities have seen simulation training as a way to advance trainee surgical skill development in a safe and easily controlled, standardised manner. The use of cost-effective, silicon anatomical models [3] to more advanced virtual reality computer program simulations [4] has been trialled in various specialities [5–7].

In 2020, the COVID pandemic challenged us to continue our annual cadaveric training workshop and ultimately led to the development of a navigated simulation-based training workshop, first introduced that year. To our knowledge, this is the first national otolaryngology specialist higher surgical training (HST) program to implement model-based FESS training into their annual training course program.

## Aims

The aim of this study was to assess the effectiveness of a novel training technique by evaluating simulation as a tool for FESS using the Kirkpatrick evaluation model.

## Methods

**Fig. 1:**
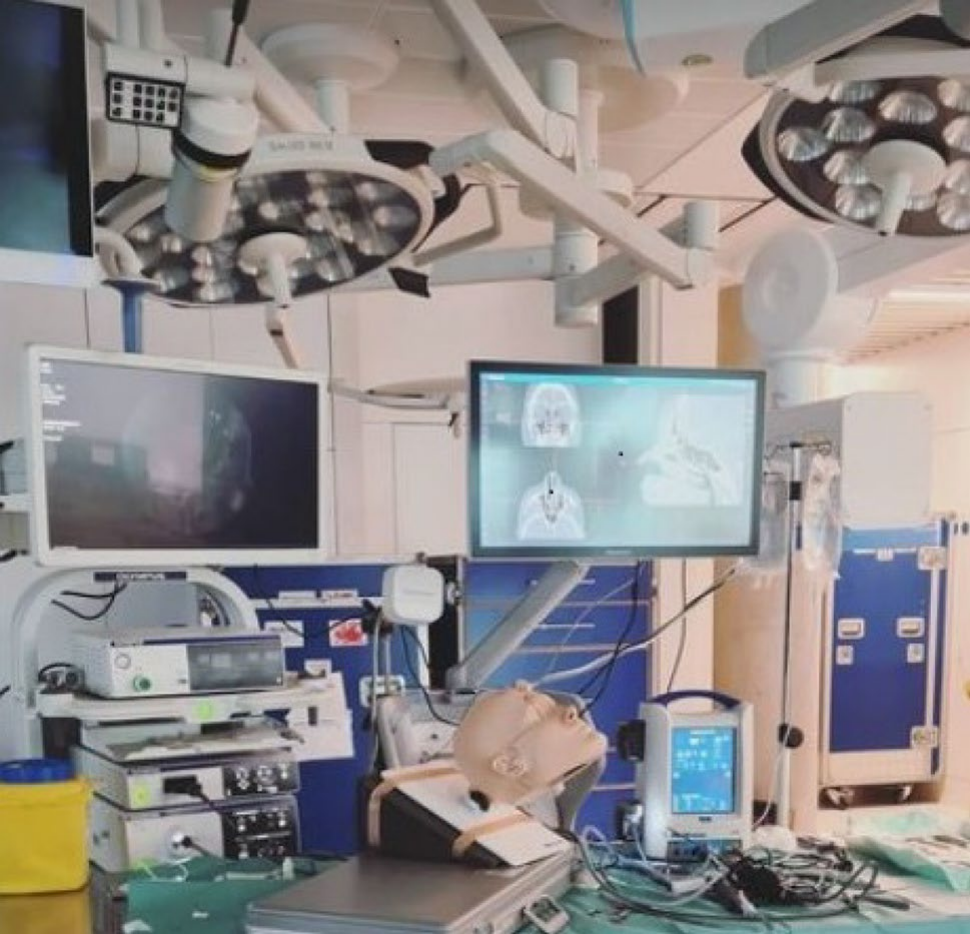
Photograph of navigated simulation training set-up

For the annual Royal College of Surgeons FESS training course for Otolaryngology HST trainees, disposable 3D printed cartridge PHACON sinus patient and trainer were used in lieu of cadaver specimens. This decision was influenced by limitations with access to cadaveric fresh frozen specimens and travel restrictions surrounding COVID-19. The course consisted of a 3-hour web-based series of introductory lectures on preparation, equipment use, safety, and ergonomics, followed by a 2-day lab-based dissection. The layout can be seen in Fig. 1, a photograph of simulation set-up using Medtronic StealthStation ENT EM Navigation System, IPC Console, and M5 Microdebrider. Phacon SN-axp simulation model and Olympus image system.

A multiple-choice questionnaire was distributed to the participants pre- and post-training course to assess anatomical and surgical learning and to ascertain trainee experience and opinions. These questionnaires were created with the assistance of the FESS course program directors and used the Kilpatrick [8] four-level evaluation model of training to synthesize outcomes into the four categories—reaction, knowledge, behaviour, and results. Qualitative results were reported as grouped data in percentage format. Paired comparisons between mean test scores, pre- and post-course, were performed using Wilcoxon’s signed rank test. Statistical analysis was performed using IBM SPSS Statistics 25.

## Results

### Pre-course demographics

A total of 21 trainees attended the course, ranging from ST3 to ST8. 19 trainees completed both the pre- and post-course components. The greatest number of cases performed was reported by an ST6 trainee. The fewest cases performed were reported by 3 trainees, ST3, ST4, and ST7. Pre-course competency in carrying out the following procedures independently were self-reported by trainees:

- 15/19 (79%) polypectomy
- 13/19 (68%) uncinectomy
- 13/19 (68%) antrostomy
- 4/19 2 (1%) ethmoidectomy
- 3/19 (16%) sphenoidotomy
- 2/19 (11%) SPA ligation
- 0% frontal sinusotomy

### Kirkpatrick 1: Reaction

Of eligible trainees, 17/21 (80%) viewed pre-course videos, 19/21 (90%) reviewed the pre-course manual, and attended the pre-course webinar. Of these, 18/19 (94%) found the pre-course material helpful.

Following the course, 20/21 (95%) agreed simulation afforded the opportunity to catch up on missed operative exposure. When asked about model-specific opinions, 19/21 (90%) agreed the models were easy to use, and 20/21 (95%) agreed the models were accurate. However, only 5/21 (25%) felt the mucosa was an accurate representation of human tissue. In particular, comments reflected this was an issue when performing septoplasty. Seventeen out of 21 (80%) agreed that cartilage and 20/21 (95%) felt that bone were realistic representations of human tissue. Additional comments suggested adding pathology and simulated bleeding would be beneficial.

Nineteen out of 21 (90%) trainees experienced a reduced operating time during COVID-19, with 18/21 (85%) specifically experiencing a reduction in exposure to FESS. Numerous trainees 12/21 (57%) commented independently that increased access would be beneficial with a request for a simulation model at each centre for training.

### Kirkpatrick 2: Knowledge

The average pre-course anatomy score was 54%, compared to 62% post-course. Using the Wilcoxon signed rank test, this was statistically significant (*Z* = 76, p0.03). Likewise, the average pre-course procedure score was 65% compared to 72% post-course (*Z* = 87, p0.03).

### Kirkpatrick 3: Behaviour

Overall, trainees had a positive view of the simulation and felt this would positively impact their future operations. In total, 20/21 (95%) agreed they could apply what they learnt in clinical practice; 19/21 (90%) agreed they had a better understanding of anatomy; 20/21 (95%) felt more comfortable operating independently following the simulation training; 20/21 (95%) felt they had safer operative techniques as a result of the simulation training. In addition, 16/21 (76%) felt their trainers were more likely to allow them to progress independently in clinical practice as a result of the simulation training, 5/21 (23%) were neutral, and 0 disagreed.

## Discussion

Surgical skill training outside of the operating theatre can be a complicated procedure. Obtaining the equipment and materials for even simple sinus surgery can be difficult. Having access to a model-based simulation would provide additional opportunities for trainees to improve their anatomical understanding and develop surgical skills. The results of our study have shown that operative exposure was not related to clinical stage, with the greatest volume of procedures > 40 reported by an ST6 trainee and one of the lowest < 5 reported by an ST7 trainee. This discrepancy in itself reflects the importance of additional surgical technical training through simulation, model, or cadaveric dissection. In addition, self-reported competency levels were very low for more advanced steps. For example, only 2/19 (11%) reported competency in sphenopalatine artery ligation, a required emergency procedure competency for any day-one consultant. From our results, the majority of participants felt they were not comfortable with all aspects of FESS and further education and training are required.

With any new training method, proper validation should be considered prior to implementation. This is outlined by benchmarks—face, construct, and content validity [9].

Face validity is established when the test meets its training objectives, in our case, simple FESS techniques. Content validity is defined when the test reflects the steps and skills used in the targeted procedure. For example, progression of preparation, identification of anatomy, and correct instrumentation for uncinectomy and middle meatal antrostomy. Construct validity is defined as the ability of the test to differentiate between novice and expert performance. The use of more complex pathology and anatomy in model cartridges will help to allow assessment of more advanced techniques.

For this study, we used the Kilpatrick training evaluation method for assessing user outcomes. The four levels of training outcomes include reaction, learning, behaviour, and results. Level one trainee reaction to or satisfaction with the training model. Level two is tangible indicators of the learning or knowledge that has been gained during training. Level three behaviour outcomes address how the knowledge is implemented, and finally, level four results provide a measure of the impact that training has had on broader organizational goals, timing or financial objectives [10]. The main limitations quoted on this method include the incompleteness of the model (disregarding preexisting organisational cultures and individual attitudes), the assumption of causality, and increasing importance as levels progress. In addition, level 4 results are particularly difficult to assess in a timely fashion in many training scenarios. For this reason, we have not been able to assess level 4 results in a setting other than trainees’ opinions as to change in their practice.

There were several limitations noted during our initial trial, many of which we have been able to address. While there is a cost associated with each cassette/model insert, this is much less than traditional cadaver cost, and as 3D printing becomes more accessible and production more readily available, this cost is likely to reduce further. As mentioned previously, the main issue identified in the initial review was the authenticity of the mucosa. Initially, it was found to be friable during attempts to elevate mucoperichondrial flaps. The second edition of the model thankfully addressed this concern introducing a thicker, more robust layer. Endoscopic sphenopalatine artery ligation can now be performed through the increased definition of both the crista ethmoidalis and arterial structure. A number of clinical pathologies were replicated, including a maxillary inverted papilloma, sphenoid mucocele, and skull base defect with CSF leak, which are novel and enhance the training experience. It is clear the future of these models is very encouraging. With developments in anatomical complexity, pathology, and individual patient replications to aid preparation of difficult cases, the potential applications are numerous. Indeed, some expert surgeons worldwide are already developing model-based courses focusing on complex frontal sinus surgery, and more will likely follow.

## Conclusions

Simulation training is an effective method of postgraduate FESS training. This has been particularly useful following reduced operative exposure in the COVID-19 era and limitations of traditional teaching methods. With continued development, we plan to make model training part of our standard annual HST program, with many more potential applications in the future.
